# Seed dispersal by the cosmopolitan house sparrow widens the spectrum of unexpected endozoochory by granivore birds

**DOI:** 10.1002/ece3.11556

**Published:** 2024-06-25

**Authors:** Guillermo Blanco, Daniel Chamorro, Ádám Lovas‐Kiss, Carolina Bravo

**Affiliations:** ^1^ Department of Evolutionary Ecology Museo Nacional de Ciencias Naturales, CSIC Madrid Spain; ^2^ Departamento de Ciencias Ambientales Universidad de Castilla‐La Mancha Toledo Spain; ^3^ Consejería de Educación Ciencia y Universidades Madrid Spain; ^4^ Department of Tisza River Research HUN‐REN Centre for Ecological Research, Institute of Aquatic Ecology Debrecen Hungary; ^5^ Institute of One Health University of Debrecen Debrecen Hungary; ^6^ Instituto de Investigación en Recursos Cinegéticos (IREC)‐(CSIC‐UCLM‐JCCM) Ciudad Real Spain

**Keywords:** animal–plant interaction, common fig, ecosystem services, internal dispersal, *Passer domesticus*, seed predation, seed survival, weeds

## Abstract

In the intricate web of plant–animal interactions, granivore birds can play a dual antagonist–mutualist role as seed predators and dispersers. This study delves into the ecological significance of the house sparrow (*Passer domesticus*) as seed disperser by endozoochory. A sample of individual droppings and faecal pools were collected from a communal roost in central Spain to examine the presence of seeds. Seed viability was determined using the tetrazolium test. Our findings revealed that around 22% of the analysed droppings contained seeds, contradicting the prevalent notion of house sparrow solely as seed predator. Viability tests demonstrated that 53.9% of the defecated seeds were viable, although it varied between plant species, including those from fleshy‐fruited common fig and five species of dry‐fruited herbs. This study challenges the traditional perspectives on the ecological role of the house sparrow, and glimpses on their contribution to seed dispersal. Understanding the nuanced roles of granivore species like the house sparrow is crucial for developing holistic conservation and management strategies in urban and agricultural landscapes. Future studies are encouraged to unravel the actual role of this cosmopolitan species as disperser of a likely broad spectrum of wild, cultivated and exotic plants.

## Graphical abstract

This study provides information that may contribute to unravel the actual role of the cosmopolitan house sparrow as disperser of a likely broad spectrum of wild, cultivated and exotic plants, an unexplored aspect that open up eco‐evolutionary and applied questions.

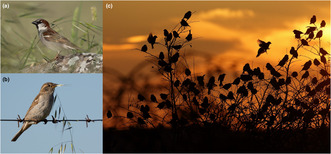

## 1 INTRODUCTION

Mutualistic relationships between animals and the plants whose seeds disperse represent pervasive ecological interactions (Bascompte & Jordano, 2014). Interaction networks have recently highlighted the dual mutualistic–antagonistic role of plant consumers and dispersers on community robustness (Montesinos‐Navarro et al., 2017). This duality represents a paradigmatic case of the conditional nature of the dispersal process, modulated by trade‐off between costs and benefits on interacting partners through multiple abiotic, biotic, ecological and population factors (Bronstein, 2015).

Despite the significant interest in understanding dispersal processes and interaction networks, many species and plant consumer guilds still lack comprehensive knowledge regarding their potential dual role in seed predation and dispersal (Fleming & Kress, 2013). In particular, generalist plant consumers like psitacids (Psittaciformes), often erroneously considered as pure seed predators, have recently been highlighted for their conditional nature as plant mutualists (Blanco et al., 2018). While several families of primary granivore birds (Phasianidae, Cacatuidae, Passeridae, Fringilidae, Emberizidae) have also been identified as potential seed dispersers (Blanco et al., 2019; Heleno et al., 2011; Holmes & Froud‐Williams, 2001; Judd, 1901; Orłowski et al., 2016; Rehling et al., 2024), comprehensive information on the species dispersed, dispersal frequency and effectiveness remains scarce for most of them and are generally not considered in seed dispersal networks.

Multiple seed dispersal syndromes, conventionally classified based on seed typology, dispersal agent and disperser action, have been criticised as insufficient and unreliable in explaining actual dispersal processes in many cases (Green et al., 2022). Specifically, endozoochory (the dispersal of seeds through ingestion and defecation by animals) has been incorrectly limited to plant species offering a reward to dispersers in the form of nutrients not contained in the seed but in the surrounding pulp of fleshy fruits (Costea et al., 2019). Among birds, fruit mashers can act as seed dispersers when inadvertently swallow tiny seeds embedded in fleshy pulp. They can also act as seed predators of the same plant species, either simultaneously or sequentially, depending on their nutritional requirements and fruiting phenological stage (Blanco et al., 2015; Fleming & Kress, 2013). In addition, primarily predators on seeds of dry‐fruited plants can also act as mashers of fleshy fruits, eventually dispersing their seeds (Fleming & Kress, 2013). These species of mainly granivorous birds (e.g. finches, Fringilidae, or sparrows, Passeridae) may represent diverse and ecologically dominant guilds, showing high richness, abundance and biomass in open environments and ecotones extensively converted into arable land (Pinowski & Kendeigh, 2012). The exploitation of crops has led to the proliferation of both weeds and crop eaters, including introduced exotic species that may disrupt ecological processes and contribute to multiple ecosystem disservices (Twigg et al., 2009).

**FIGURE 1:**
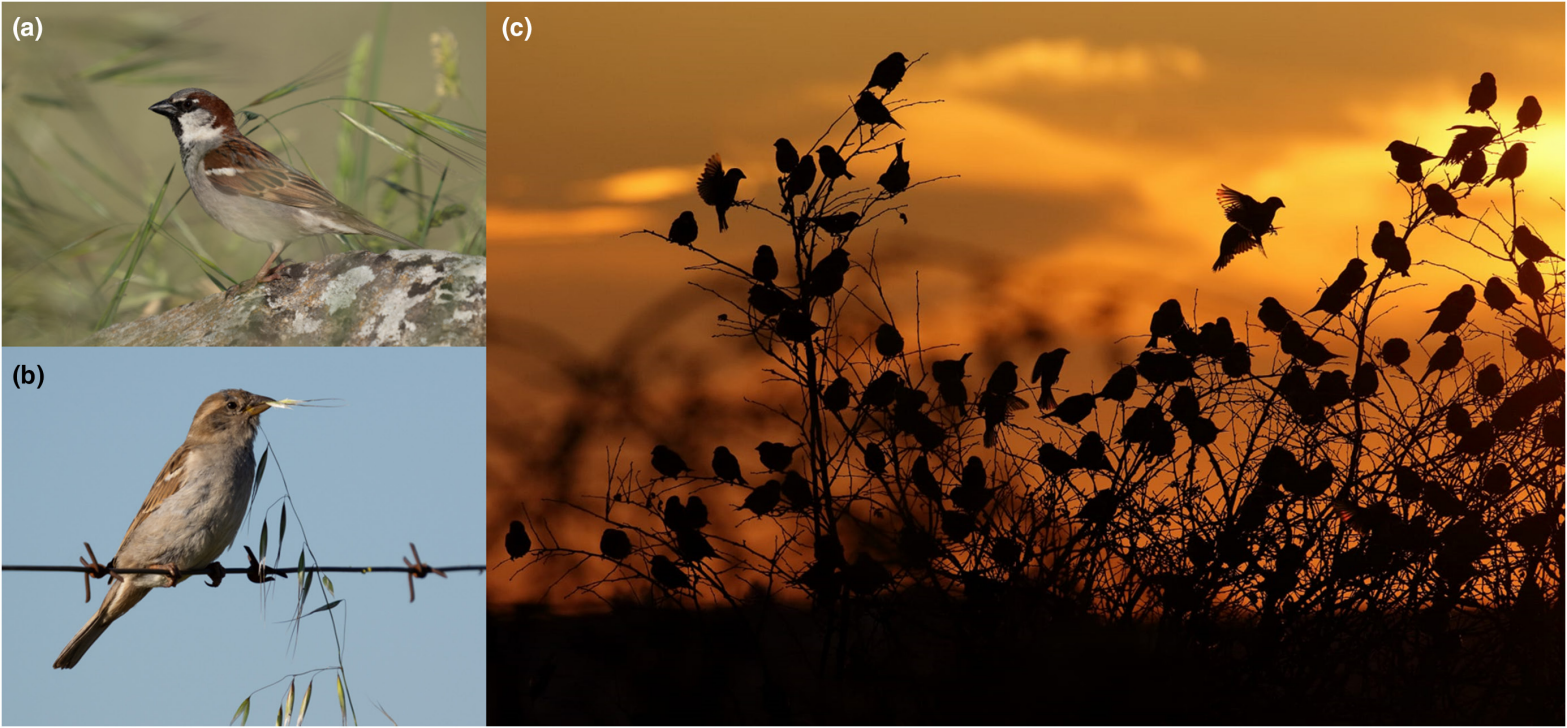
(a) An adult male and (b) female house sparrow (*Passer domesticus*) (photos by Juan C. Fernández Mata). (c) A flock of house sparrows just before entering to a communal roost (photo by Alfredo López Hernangómez).

Among invasive granivores birds demonstrating notable success in their expansion and numerical proliferation, the house sparrow (*Passer domesticus*) (Figure 1) stands as a species that has colonised practically all regions of the world, adapting to multiple environments, especially urban and cultivated areas (Anderson, 2006). The global spread of house sparrows can influence their ecological roles in non‐native regions as a potential competitor able to displace native species in nesting and foraging contexts (Avery & Lockwood, 2017). Despite its abundance worldwide, there is a growing concern regarding the decline in its native range over the past decades (De Laet & Summers‐Smith, 2007). A comprehensive study on the feeding habits of this species revealed the significant role of seeds from cultivated and weed species in their diet (Anderson, 2006). Early reports suggest that seeds of weeds can pass through the digestive tract in a viable condition in this and other sparrow species (Judd, 1901; Southern, 1945), but specific data on their potential role as a seed disperser remains lacking. It is of paramount importance to comprehend the ecological function of this cosmopolitan and abundant species in communities of open, agricultural and urban environments. This is due to its prominence in ecological interaction networks of such socio‐economically significant environments.

Here, we evaluate the potential role of the house sparrow as an endozoochoric seed disperser of the plants it consumes. Given its generalist diet, it is expected that by acting as a fruit masher, this species could disperse tiny seeds of fleshy‐fruited plants. By feeding on seeds of dry‐fruited crops and herbs, their role as a seed disperser is more counterintuitive. Although seeds are expected to be cracked for efficient digestion, a proportion of these seeds could remain intact and pass through the gut to be potentially defecated in a viable form. Confirmation of endozoochory in this species could elucidate its pivotal role as a provider of both ecosystem services and disservices through this dispersal process. It could potentially include a wide variety of wild and cultivated plants throughout its extensive native and introduced range.

## 2 MATERIALS AND METHODS

During the summer of 2016 and 2017, droppings from a communal roost of house sparrows were collected in Torrelodones, Madrid, central Spain. The roost was exclusively used by this species, which was confirmed by frequent observations, especially the afternoon before the collection of faeces. The number of house sparrows using the roost ranged between 40 and 60 individuals depending on the time of sampling. The study area comprises built‐up, gardens and open mixed oak‐pine forests in a residential area (Molina, 2019). In this area, the sparrows feed on seeds from cultivated and wild herbs, tree and shrub fruits and seeds, as well as food scraps from the human population. The roost was established on climbing plants (*Hedera helix*, *Parthenocissus quinquefolia*) adjacent to a building.

A transparent strip plastic material was placed on the ground under the roosting perches of the sparrows the day before each sampling to facilitate the overnight collection of their droppings. We collected a random sample of entire droppings in the morning, just after the sparrows left the roost. Each dropping was stored individually in a paper envelope. Non‐adjacent faeces were selected to avoid sample duplication from the same individual on each sampling day. In addition, given the difficulty of individualising each dropping due to their accumulation in certain parts of the roost floor, and to increase the probability of finding seeds from more species, pools of droppings were also collected randomly over different areas of the roost, and stored together in the same way as the individual samples. The droppings were dried in an oven (at 25°C) and kept at room temperature until analysis. In total, individual droppings were collected during July (*n* = 30) and August (*n* = 42) of 2016. Pools of drooping were collected in October of 2016 (*n* = 2), July of 2017 (*n* = 1), and August of 2017 (*n* = 3).

Individual droppings and pools were weighed, and disaggregated with the help of tweezers under a binocular microscope (20×) to determine the presence of seeds. Recovered seeds were measured, photographed and identified based on their size and external features. Seed viability was determined with the tetrazolium test (Moore, 1985). The test was performed by a thin slice off from distal end of each seed made before incubating them in a 1% solution of 2,3,5‐triphenyl tetrazolium chloride for 48 h under dark conditions. Viable seeds were all those whose embryos were stained red, while embryos partially stained or unstained seeds were considered corresponding to non‐viable seeds.

## 3 RESULTS

Overall, we recovered 227 seeds from six species and five families. From individual droppings (*n* = 72), a total of 93 seeds of four species were recovered, representing an occurrence frequency of 21.8%. From pools of droppings (*n* = 6), a total of 134 seeds of four species (two species not found in individual droppings) were recovered, amounting 14.3 g of faeces, which represents 9.5 seeds/g of faeces in the pooled samples. The occurrence frequency, mean number of seeds per dropping, and weight of faeces in pools for each recovered species is shown in Table 1.

**TABLE 1:** Number and percentage of house sparrow droppings and pools of drooping with intact seeds of each species.

Plant species	Seed size, mm (*n*)	Seeds recovered	*n* individual droppings with seeds (%)	Mean ± SD seeds/individual dropping (range)	Seeds/g faeces pools	Viable/tested seeds (% viability)
*Ficus carica* (Moraceae)	2.09 × 1.37 (10)	169	7 (9.7)	8.7 ± 9.6 (1–28)	10.20	66/160 (41.3)
*Geranium* sp. (Geraniaceae)	1.79 × 1.04 (11)	48	9 (12.5)	3.2 ± 3.5 (1–12)	2.39	45/47 (95.7)
Unidentified Fabaceae	1.63 × 1.18 (2)	2	1 (1.4)	2	‐	2/2 (100)
*Rumex* sp. (Polygonaceae)	1.50 × 0.70 (1)	1	1 (1.4)	1	‐	1/1 (100)
*Digitaria sanguinalis* (Poaceae)	1.72 × 0.70 (6)	6	‐	‐	0.63	0/6 (0.0)
Unidentified Poaceae	1.66 × 0.78 (1)	1	‐	‐	0.09	1/1 (100)
Total		227	15 (21.8)	1.3 ± 4.2 (1–28)	13.30	117/217 (53.9)

*Note*: Seed size (mean length × width) was indicated for each species. *n* = sample size.

Seeds were consistently found in all sampling months, with monthly variations in the recovered species. Seeds of common fig (*Ficus carica*) were recovered in every monthly sampling of individual droppings (10.0% in July 2016, *n* = 30, and 9.5% in August 2016, *n* = 42), as well as in dropping pools (25.7 seeds/g in October 2016, 2.5 seeds/g in July 2017, and 1.4 seeds/g in August 2017). *Geranium* sp. seeds were also recovered in every monthly sampling of individual droppings (3.3% in July, *n* = 30, and 19.0% in August, *n* = 42) and pools (1.5 seeds/g in October 2016, 0.3 seeds/g in July 2017, and 2.4 seeds/g in August 2017). A single *Rumex* sp. seed and two seeds of an unidentified Fabaceae were found in a single dropping each in July 2016, but not in pools in July 2017. Seeds of *Digitaria sanguinalis* and an unidentified Poaceae were recovered only in pools from October. Most droppings containing seeds comprised a single species (80.0%, *n* = 15), while the remaining samples contained concurrent seeds of both *Ficus carica* and *Geranium* sp.

Of the recovered seeds, 53.9% exhibited viability (pooling all seeds), with high variability among species. Most of *Geranium* sp. seeds were viable (95.7%), while those of common fig had lower viability (41.3%). The viability of the recovered seeds of each species is shown in Table 1.

## 4 DISCUSSION

This study demonstrates that the house sparrow can act as a legitimate endozoochorous seed disperser, maintaining viability and propagating several of the plant species on which it feeds. This species has been previously considered as a primary seed predator often acting as a pest of cultivated grain. To our knowledge, previous research has often overlooked the examination of their droppings to confirm the fate of consumed seeds – whether they are crushed and digested, or potentially viable upon defecation. Approximately 22% of the analysed droppings contained entire seeds, generally smaller than 2 mm in diameter, from six plant species. While the proportion of intact seeds defecated among those ingested was unknown, the rate of droppings containing viable seeds is remarkable for a primary seed‐eater (Fleming & Kress, 2013).

The viability of the recovered seeds varied among plant species, with about half of the total number of seeds examined demonstrating viability according to the tetrazolium test. Despite the limitations of our study, focusing on a single location and a limited number of sampled individuals from a single communal roost, our results confirmed seed dispersal in two different seasons, and across all the sampled months. Monthly variations in the richness and abundance of dispersed seeds from different species suggest the potential for broader year‐round sampling across different habitats and regions within the house sparrows extensive range to provide a more comprehensive understanding of its seed dispersal capabilities.

Among the recovered seeds, we found those of the fleshy‐fruited common fig, a wild and cultivated plant in the Mediterranean. When feeding on the fruit pulp, the house sparrow behaves as a typical endozoochorus disperser by defecating seeds (about 40% viable) after gut passage (Fleming & Kress, 2013). Like many other fruit mashers, this species seems to inadvertently ingest the tiny seeds embedded in small pecked fragments of the carbohydrate‐rich pulp of the fig fruit. In general, fruit mashers have been considered less efficient seed dispersers than fruit gulpers, as a proportion of seeds may be crushed in the process of mandibulating pulp, or because these fruit consumers primarily target seeds (Fleming & Kress, 2013). This dispersal mechanism could be widespread among house sparrows, given their high abundance worldwide, and because of their generalist feeding habits on wild and cultivated fleshy‐fruited plants (Anderson, 2006). Dispersal by epizoochory of tiny seeds embedded in pulp has been recorded for multiple masher species (Fleming & Kress, 2013; Hernández‐Brito et al., 2021). This is also possible in the house sparrow, although its confirmation requires specific studies involving the capture of specimens to determine the presence of seeds attached to the beak.

The remainder of the recovered seeds belonged to several families of dry‐fruited herbs. Among them, the seeds of *Geranium* sp. showed a higher occurrence and number per dropping, along with almost absolute viability rate. Seed dispersal of these herbs is generally assumed to occur abiotically or by ectozoochory (Green et al., 2022; Nathan et al., 2008). For example, in *Geranium* species, the capsule‐shaped fruit opens when ripe, dispersing the seeds over short distances (Yeo, 1984). Dispersal by endoozocory of these seeds by a granivore bird whose beak is adapted to crack seeds to digest them seems counterintuitive since the seeds must be ingested whole and defecated undamaged to maintain their viability (Twigg et al., 2009). This dispersal mechanism has generally been overlooked, despite that it could potentially lead to the long‐distance spread of herbs that are typically associated with dispersal through abiotic agents (Green et al., 2022). Defecation of viable seeds could be due to digestive trade‐offs driven by seasonal changes in diet and its processing for nutrient assimilation (Herrera, 1984; Kleyheeg et al., 2015; Soons et al., 2016). Birds exhibited particularly brief retention time within the digestive tract, and consequently the seed has minimal contact with stomach acids, which also contributes to its long‐term viability (Klasing, 1999). This process necessarily involves swallowing the whole seed, which may be modulated by the consumer predation risk or competition for food requiring quick feeding in both cases. This could favour the ingestion of entire seeds and their complete transit through the digestive tract. It implies that apparently suboptimal feeding behaviours can be associated with enhanced long‐distance dispersal. In addition, the shape and coat features of seeds of particular plant species could enhance swallowing or inhibit their digestion, which require further research for the assessment of the dispersal process by granivore birds (Twigg et al., 2009).

In conclusion, this study contributes to previous evidence supporting the endozochorous seed dispersal by granivorous birds, generally considered only as seed predators. It is recommended that the house sparrow and other granivores be included in the study of seed dispersal, as it could be shown that these species play an important role in endozoochoric dispersal, while also maintaining seed viability. Furthermore, in the context of future research projects, it is recommended that sampling be conducted throughout the year, as the abundance and richness of seeds varies depending on the supply of fruits during the year. Ecosystem services and disservices arising from the dual roles of the house sparrow in predating and dispersing crops and weeds become notably significant in quantitative terms, given its widespread range and huge abundance worldwide. Future studies are encouraged to unravel the actual role of this cosmopolitan species as a predator and disperser of a likely broad spectrum of wild, cultivated and exotic plants.

## AUTHOR CONTRIBUTIONS

**Guillermo Blanco:** Conceptualization (equal); data curation (equal); formal analysis (equal); investigation (equal); methodology (equal); project administration (equal); supervision (equal); validation (equal); writing – original draft (equal). **Daniel Chamorro:** Conceptualization (equal); formal analysis (equal); methodology (equal); writing – review and editing (equal). **Ádám Lovas‐Kiss:** Conceptualization (equal); formal analysis (equal); methodology (equal); writing – review and editing (equal). **Carolina Bravo:** Conceptualization (equal); formal analysis (equal); investigation (equal); methodology (equal); validation (equal); writing – review and editing (equal).

## FUNDING INFORMATION

No fund was available.

## CONFLICT OF INTEREST STATEMENT

We declare we have no competing interests.

## Data Availability

The data set used in the current study is available at https://zenodo.org/records/11216360.
